# Serum uric acid and nonalcoholic fatty liver disease

**DOI:** 10.3389/fendo.2024.1455132

**Published:** 2024-11-28

**Authors:** Jia Fan, Dongxu Wang

**Affiliations:** Department of Gastroenterology, Shengjing Hospital of China Medical University, Shenyang, China

**Keywords:** nonalcoholic fatty liver disease, serum uric acid, xanthine oxidase, oxidative stress, NLRP3 inflammasome, lipid metabolism, insulin resistance

## Abstract

Nonalcoholic fatty liver disease (NAFLD) is characterized by over 5% hepatic fat accumulation without secondary causes. The prevalence of NAFLD has escalated in recent years due to shifts in dietary patterns and socioeconomic status, making it the most prevalent chronic liver disease and a significant public health concern globally. Serum uric acid (SUA) serves as the end product of purine metabolism in the body and is intricately linked to metabolic syndrome. Elevated SUA levels have been identified as an independent risk factor for the incidence and progression of NAFLD. This paper reviews the relationship between SUA and NAFLD, the underlying mechanisms of SUA involved in NAFLD, and the potential benefits of SUA-lowering therapy in treating NAFLD. The aim is to raise awareness of SUA management in patients with NAFLD, and to encourage further investigation into pharmacological interventions in this area.

## 1 Introduction

Nonalcoholic fatty liver disease (NAFLD) is a clinicopathological condition characterized by over-accumulation of fat in the liver, defined as steatosis in over 5% of hepatocytes, in the absence of alcohol consumption and other definitive factors causing liver damage (1, 2). The prevalence of NAFLD has been steadily rising due to changes in lifestyle and dietary patterns, making it the most common chronic liver ailment globally (3, 4). The global prevalence of NAFLD is 30.1% (5), with Asian countries reporting a prevalence of 29.6% (6). NAFLD not only has the potential to progress to cirrhosis and liver cancer but is also linked to cardiovascular and cerebrovascular diseases, peripheral vascular diseases, diabetes mellitus, cholelithiasis, and other conditions, as well as an increased risk of various malignant tumors such as colorectal, breast, and pancreatic cancers (7–9). NAFLD is a serious threat to the health of human beings, and it has become a major global concern (10, 11). Given the widespread use of the term NAFLD in existing literature, despite recent proposals to rename it as metabolic-associated fatty liver disease (MAFLD) (12), this review will continue to refer to it as NAFLD.

Uric acid (UA) is the final product of purine compound breakdown in the liver (13), with xanthine oxidase (XO) playing a crucial role in its production by catalyzing the oxidation from hypoxanthine to xanthine and then to UA (14). Abnormalities in UA metabolism are associated with several chronic systemic diseases like hypertension, atherosclerosis, diabetes mellitus, and dyslipidemia (15–17). The relationship between serum uric acid (SUA) levels and NAFLD severity has gained attention in recent years, with SUA being a significant factor independently correlated with the severity of NAFLD independently of other metabolic markers (18). Studies have reported a 21% increase in NAFLD risk for every 1 mg/dL rise in SUA levels (19), with hyperuricemia further elevating the risk of significant liver fibrosis in NAFLD patients (20).

In order to gain a deeper comprehension of the correlation between SUA and NAFLD, this review aims to synthesize current research findings to aid in the management of SUA levels in NAFLD patients.

## 2 Relationship between SUA and NAFLD

A Meta-analysis has shown a pooled odds ratio of 1.88 in NAFLD patients with higher SUA levels compared to those with lower levels, with SUA levels being associated with NAFLD across various subgroups regardless of study quality, study design, sample size, age, gender, or country (21). Elevated SUA levels are also linked to the severity and progression of NAFLD, with studies indicating a strong correlation between SUA levels and the degree of steatosis, inflammation of the lobules, cirrhosis development, and elevated liver enzymes in NAFLD patients (22, 23). While obesity is a known risk factor for NAFLD (24), increased SUA levels in non-obese individuals significantly heighten the risk of NAFLD, surpassing that in obese patients with normal SUA levels (25). **Table 1** provides an overview of relevant studies on the relationship between SUA on NAFLD (18, 22, 25–61).

**Table 1:** Studies on the relationship between SUA on NAFLD.

Size	Types of studies	Country	Main Findings. (Reference)	Author, Year
3,499	cross-sectional study	China	SUA level demonstrated a positive correlation with the prevalence of NAFLD. A regression equation was developed to predict NAFLD, expressed as follows: The proposed regression formula = 0.032 * WC + 0.303 * BMI + 0.478 * natural logarithm of glutamyl transpeptidase + 1.301 * natural logarithm of triglyceride + 0.002 * SUA - 18.823. When utilizing a cutoff value of 13.3, the model exhibited a sensitivity of 89.2% and a specificity of 78.4% (26).	Ding Y, 2023
1,343	cross-sectional study	Korea	SUA levels strongly correlated with fatty liver indices. SUA concentrations in individuals diagnosed with NAFLD and exhibiting abnormal LFT outcomes were notably elevated compared to those without NAFLD and abnormal LFT findings (27).	Park H, 2022
4,554	cross-sectional study	China	SUA thresholds were of ≥478 µmol/L and ≥423.5 µmol/L for severe steatosis in male and female MAFLD patients. NAFLD patients with higher SUA levels exhibited greater liver fat accumulation compared to those with lower SUA levels. Even among lean/normal-weight patients with NAFLD, higher SUA levels were associated with an increased likelihood of severe steatosis (28).	He J, 2022
3,311	cross-sectional study	China	Increased SUA levels were identified as a facilitating factor for the development of NAFLD after accounting for relevant confounding variables (OR = 2.44). The risk of NAFLD exhibited a linear relationship with rising SUA levels (29).	Wang M, 2022
27,009	cohort Study	China	SUA exhibited a positive correlation with the occurrence of NAFLD, particularly among female and non-obese individuals, and was also linked to an increased likelihood of potential advancement of newly diagnosed NAFLD (30).	Tang Y, 2022
454	cross-sectional study	Italy	There was a significant correlation between hyperuricaemia and NAFLD (31).	Catanzaro R, 2022
2,809	cross-sectional study	China	In patients with type 2 diabetes mellitus, an increased SUA level was identified as a standalone risk factor for the occurrence of NAFLD following accounting for other relevant variables (32).	Hu Y, 2021
139,170	cross-sectional study	China	Individuals with MAFLD had a notably higher prevalence of hyperuricemia compared to those without MAFLD (45.0% vs. 16.8%) (33).	Chen YL, 2021
400	cross-sectional study	China	The levels of SUA exhibit a significant and autonomous correlation with the occurrence of NAFLD. SUA levels can serve as a valuable indicator for identifying non-obese individuals with type 2 diabetes who are at an elevated risk for developing NAFLD (34).	Cui Y, 2021
4,323	cross-sectional study	China	There was a significant positive correlation and dose-response pattern between SUA levels and the incidence of NAFLD in postmenopausal individuals who were not obese. Elevated SUA levels may serve as a potential prognostic indicator for NAFLD in non-obese postmenopausal women (35).	Bao T, 2020
3,822	prospective cohort study	China	Elevated SUA levels that exhibit an increasing trend pose a risk factor for NAFLD. This association demonstrates a dose-response relationship that remains consistent across different age groups, genders, and individuals with abdominal obesity (36).	Ma Z, 2020
2,832	prospective cohort study	China	NAFLD is directly associated with higher levels of SUA, with elevated SUA concentrations serving as a potential standalone indicator for the development of NAFLD. The established SUA thresholds indicative of NAFLD risk are as follows: ≥288.5 μmol/L for the general population, ≥319.5 μmol/L for males, and ≥287.5 μmol/L for females (37).	Wei F, 2020
113	cross-sectional study	Indonesia	Hyperuricemia was identified as a distinct risk factor associated with the development of substantial liver fibrosis (OR = 2.501) (38).	Sandra S, 2019
100	cross-sectional study	Pakistan	NAFLD associated with SUA levels (39).	Abbasi S, 2019
367	cross-sectional study	Turkey	UA serves as an uncomplicated, non-intrusive, cost-effective, and valuable indicator that can potentially forecast the presence of steatosis in individuals with NAFLD. The identified threshold level for SUA was determined to be 4.75 mg/dl, exhibiting a sensitivity of 45.8% and a specificity of 80.3% (40).	Oral A, 2018
856	observational cohort study	China	The likelihood of NAFLD rose in correlation with elevated SUA levels, with SUA level identified as a standalone risk factor for NAFLD, exhibiting a RR value of 1.654 (41).	Bai JX, 2018
7,569	cross-sectional study	China	There was a positive correlation between SUA levels and the prevalence of NAFLD, with a slightly stronger association observed in women compared to men. Furthermore, a significant combined effect of SUA levels and serum ALT levels on NAFLD prevalence was noted in all participants, with a slightly greater impact observed in men than in women (42).	Yang H, 2018
826	retrospective cohort study	China	In contrast to individuals with normal UA levels, those with hyperuricemia exhibited notably elevated levels of total cholesterol, creatinine, triglycerides, and AST. Furthermore, individuals with hyperuricemia demonstrated a significantly reduced probability of NAFLD remission compared to those with normouricemia (RR = 0.535) (43).	Yang C, 2018
95,924	cross-sectional study	China	Increased SUA concentrations were found to be correlated with a heightened likelihood of lean NAFLD. Lean individuals with hyperuricemia exhibited an OR of 1.718 for the presence of NAFLD, following adjustments for additional metabolic disorders. The diagnostic accuracy, as indicated by the AUC, for identifying mild NAFLD using SUA was 0.70, while the AUC for detecting moderate to severe NAFLD based on SUA was 0.78 (44).	Zheng X, 2017
1,006	cross-sectional study	China	Elevated SUA levels were linked to a higher risk of NAFLD in both males and females, with OR of 2.645 and 1.962. A notable gender disparity was observed in the association between hyperuricemia and NAFLD, with a statistically significant difference found in males compared to females (45).	Yu XL, 2017
2,383	retrospective cohort study	China	The prevalence of NAFLD was found to be higher in individuals with elevated SUA levels compared to those with normal levels (29.0% vs. 12.9%). Hyperuricemia at baseline was significantly linked to an increased risk of developing NAFLD in non-obese individuals. Furthermore, the impact of hyperuricemia on NAFLD risk was more pronounced in females (RR = 2.138) than in males (RR = 1.435) (46).	Yang C, 2017
4,098	cross-sectional study	China	Non-obese individuals exhibit a greater susceptibility to NAFLD with elevated SUA levels compared to obese individuals. Furthermore, the advancement of inflammation in NAFLD is linked to elevated SUA levels in non-obese individuals (25).	Liu J, 2017
841	prospective cohort study	China	SUA levels exhibited an inverse correlation with the remission of NAFLD. Individuals with elevated SUA concentrations at the outset demonstrated reduced rates of NAFLD remission (47).	Zhou Z, 2016
158	cross-sectional study	China	SUA level demonstrated a positive correlation with the extent of steatosis, with a correlation coefficient of 0.177. Patients with hyperuricemia exhibited a higher prevalence of severe lobular inflammation (lobular inflammation score ≥2) compared to those with normal SUA levels (75% vs. 52.7%). Individuals with NAFLD in the hyperuricemic groups displayed a greater incidence of non-alcoholic steatosis (≥5) in comparison to those in the normal SUA groups(48.8% vs. 31.1%). Hyperuricemia was identified as an independent factor associated with advanced lobular inflammation (OR = 2.79) (22).	Huang Q, 2016
110	cross-sectional study	Bangladesh	Elevated SUA levels have been found to be closely linked with NAFLD, with this relationship appearing to be influenced by IR in individuals with prediabetes (48).	Hossain IA, 2016
118	cross-sectional study	Itady	SUA was identified as a significant independent predictor of non-alcoholic steatohepatitis and its specific histological manifestations, particularly fibrosis (49).	Ballestri S, 2016
4,305	cross-sectional study	China	LFC accumulation was found to be linked to a rise in the occurrence of hyperuricemia and elevated SUA levels within a population residing in community settings. An LFC exceeding 10% is correlated with an increased likelihood of developing hyperuricemia (50).	Lin H, 2015
60,455	multicenter Study: cross-sectional study and prospective study	China	A gender-specific SUA concentration was found to be linked with NAFLD in a manner independent of other factors. Moreover, the correlation between SUA levels and NAFLD was notably more pronounced in females compared to males (51).	Wu SJ, 2015
6,967	cross-sectional study	USA	The incidence of NAFLD was notably greater among individuals with hyperuricemia in comparison to those without (33.8% vs. 14.7%). Those with hyperuricemia exhibited a higher occurrence of elevated liver enzymes in contrast to those without (AST 8.9% vs. 3.0%; ALT 9.6% vs. 4.7%) (52).	Shih MH, 2015
21,798	cross-sectional study	China	The risk of NAFLD was notably higher in individuals with elevated SUA levels. Moreover, a significant correlation was observed between SUA levels and prehypertension in terms of the risk of NAFLD (53).	Liang J, 2015
528	cross-sectional study	China	Elevated SUA levels, even when falling within the normal range, were positively and independently correlated with the occurrence of hepatic steatosis in postmenopausal Chinese women with a normal BMI (54).	Liu PJ, 2014
242	cross-sectional study	Turkey	Hyperuricemia was frequently observed in individuals with NAFLD and was linked to the presence of initial histological manifestations, such as hepatocellular ballooning, in this significant clinical context (55).	Sertoglu E, 2014
1,440	epidemiological cohort study	China	In Chinese males, there is a notable correlation between elevated SUA levels and NAFLD. Moreover, within individuals diagnosed with NAFLD, indicators of liver impairment, such as heightened ALT levels in conjunction with a genetic predisposition (specifically the Met196Arg variant in TNFRSF1B (rs1061622)), are linked to elevated SUA concentrations associated with inflammatory processes (56).	Xie Y, 2013
10,605	comparative Study	China	The incidence of NAFLD was found to be higher with elevated SUA levels, with a more notable correlation observed in Uyghur individuals compared to Han individuals (OR = 3.279 and 3.230, respectively) (57).	Cai W, 2013
10,732	cross-sectional study	USA	An increased serum uric acid (SUA) level was found to be linked with non-alcoholic fatty liver disease (NAFLD) diagnosed through ultrasound in a sample of nondiabetic adults representative of the United States population. Furthermore, a positive correlation was observed between rising uric acid levels and the severity of NAFLD as determined by ultrasonography (18).	Sirota JC, 2013
9,019	cross-sectional study	Korean	Elevated SUA levels, even when falling within the normal range, were found to be independently linked to the occurrence of NAFLD (58).	Hwang IC, 2011
5,741	cohort study	Korean	Elevated SUA levels were identified as a significant independent risk factor for the development of NAFLD as determined by ultrasonography. After adjusting for relevant variables, the hazard ratio for individuals with hyperuricemia compared to those with normouricemia was 1.29 (59).	Ryu S, 2011
6,890	prospective study	China	The rise in SUA levels was a significant independent predictor of heightened risk for developing NAFLD, with the likelihood of NAFLD occurrence rising in correlation with escalating baseline SUA levels (60).	Xu C, 2010
54,325	cross-sectional study	China	There was a significant correlation between gout and the risk of NAFLD. Furthermore, a proportional connection was observed between SUA levels and NAFLD risk in individuals both with and without gout (61).	Kuo CF, 2010
8,925	cross-sectional study	China	The frequency of NAFLD was notably greater among individuals with hyperuricemia compared to those without hyperuricemia, with rates of 24.75% and 9.54% respectively. Moreover, the prevalence of NAFLD exhibited an upward trend with escalating SUA levels (62).	Li Y, 2009

NAFLD, nonalcoholic fatty liver disease; SUA, serum uric acid; LFT, liver function test; MAFLD, metabolic-associated fatty liver disease; UA, uric acid; OR, odds ratio; WC, waist circumference; RR, relative risks; BMI, body mass index; AST, aspartate transaminase; AUC, area under the curve; ALT, alanine aminotransferase; AUC, area under curve; LFC, liver fat content; USA, United States of America; IR, insulin resistance; BMI, body mass index. *, multiply.

## 3 Underlying mechanisms of SUA involved in NAFLD

**Figure 1:**
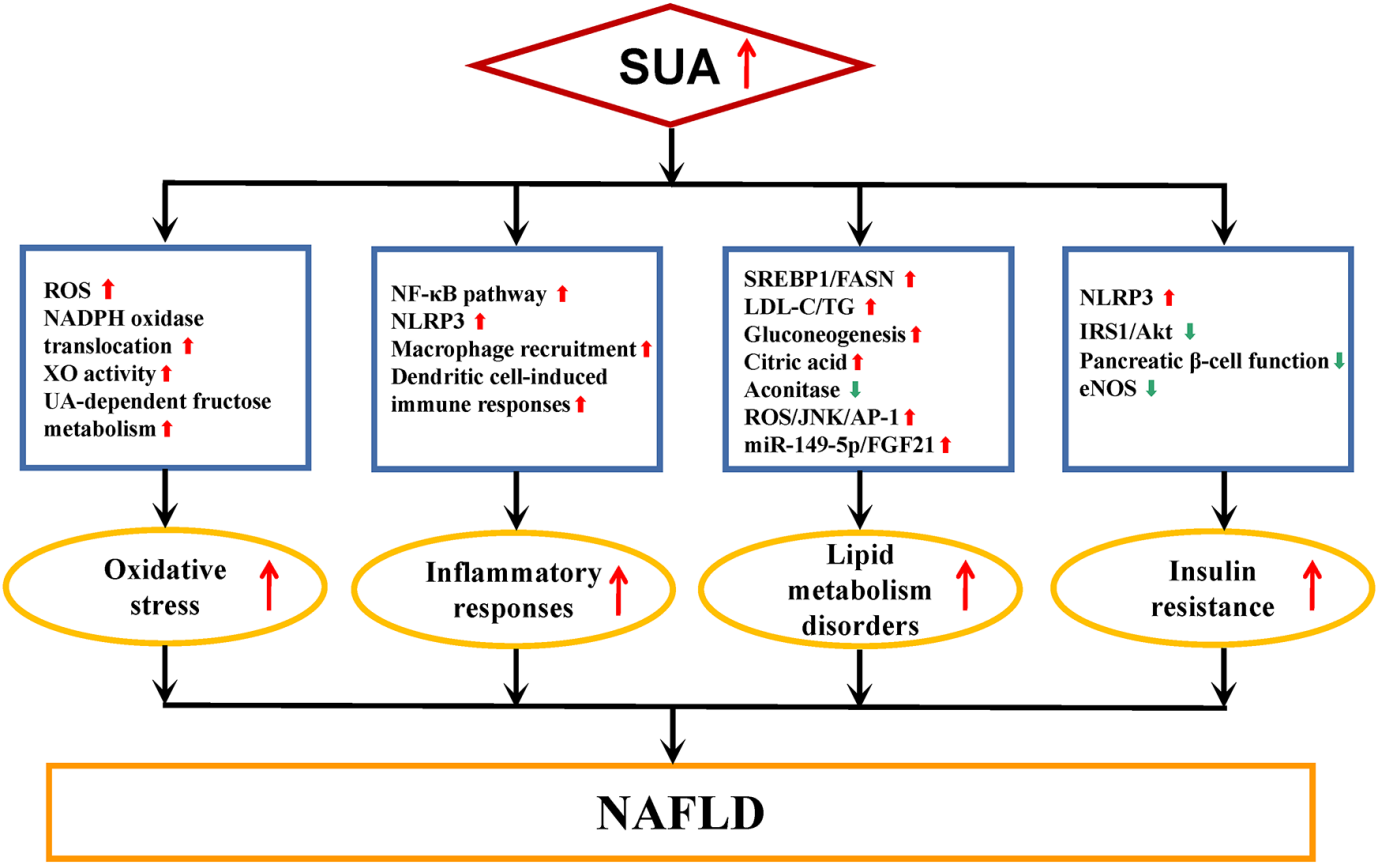
Underlying mechanisms of SUA involved in NAFLD. NAFLD, nonalcoholic fatty liver disease; SUA, serum uric acid; ROS, reactive oxygen species; UA, uric acid; NADPH, nicotinamide adenine dinucleotide phosphate; XO, xanthine oxidase; NF-κB, nuclear factor kappa-B; NLRP3, nucleotide-binding oligomerization domain-like receptor family pyrin domain containing 3; SREBP1, sterol regulatory element binding protein 1; FASN, fatty acid synthase; LDL-C, low-density lipoprotein cholesterol; TG, liver fat content; JNK, c-Jun N-terminal kinase; AP-1, activator protein-1; FGF21, fibroblast growth factors 21; IRS1, insulin receptor substrate 1; Akt, protein kinase B; eNOS, endothelial nitric oxide synthase. Red arrows, up-regulation of expression or enhanced activity. Green arrows, down-regulation of expression or reduced activity.

The pathogenesis of NAFLD has transitioned from the “two-hit” theory to the “multiple hit” hypothesis, which suggests that various factors collectively contribute to the development of NAFLD in genetically susceptible individuals (63, 64). These factors include steatosis, inflammation, oxidative stress (OS), metabolic dysfunction, and insulin resistance (IR) (63, 64). The precise mechanisms through which SUA is involved in NAFLD remain incompletely understood. SUA plays a role in the onset and progression of NAFLD through processes such as OS, inflammatory responses, disturbances in lipid metabolism, and IR, as shown in **Figure 1**.

### 3.1 Oxidative stress

OS is an important etiopathogenesis of NAFLD (65). Elevated levels of reactive oxygen species (ROS) under OS can lead to dysfunction in mitochondria and endoplasmic reticulum, as well as reduced antioxidant defenses in liver cells, resulting in inflammation, cell death, and fibrosis during NAFLD progression (66). Hepatocytes exposed to UA has been linked to mitochondrial OS mediated by the translocation of nicotinamide adenine dinucleotide phosphate (NADPH) oxidase (67). UA also enhances fat synthesis in hepatocytes by facilitating the transfer of NADPH oxidase subunit 4 to mitochondria, thereby increasing superoxide production (68). Xanthine oxidase (XO), the rate-limiting enzyme enzyme for UA production, generates ROS during catalyzing the oxidative hydroxylation of hypoxanthine and xanthine to produce UA (69). SUA levels can serve as an indicator of XO activity in NAFLD (70). In addition, 70% of fructose in the human body is metabolized by the liver, and fructose-rich diets can exacerbate NAFLD (71). Fructose can exacerbate NAFLD by promoting hepatic fat accumulation through both direct triglyceride (TG) synthesis from fructose metabolism and an uric acid-dependent pathway via mitochondrial OS (72).

### 3.2 Inflammatory responses

Inflammation is a fundamental component of NAFLD pathophysiology and is present throughout the disease progression (73). UA is a potent inflammatory inducer, capable of upregulating the expression of various inflammation markers in a dose-dependent manner (74). It may also trigger the activation of pro-inflammatory signaling pathways, such as nuclear factor kappa-B, leading to the expression of inflammatory molecules and exacerbating the inflammatory response in hepatocytes (74). The nucleotide-binding oligomerization domain-like receptor family pyrin domain containing 3 (NLRP3) inflammasome, a multiprotein complex involved in recognizing pathogens and molecular patterns, plays a crucial role in obesity, IR, and NAFLD progression (75–77). UA, as an injury-related molecular pattern, stimulates macrophage recruitment (78, 79), which then expresses NLRP3 inflammasome and generate significant quantities of IL-1β (80, 81), leading to chronic inflammation in hepatocytes. UA also exacerbate hepatic inflammation by inducing immune responses through dendritic cells (82).

### 3.3 Lipid metabolism

The onset of NAFLD is linked to disruptions in lipid metabolism (83). UA plays a role in regulating fatty acid synthase (FASN) through the sterol regulatory element binding protein 1 (SREBP1) signaling pathway, resulting in the accumulation of free fatty acids and compromised energy metabolism in HepG2 cells (84). Additionally, UA triggers the activation of SREBP-1c via endoplasmic reticulum stress, promoting lipid synthesis in hepatocytes, which in turn exacerbates intracellular fat accumulation and lipid degeneration in hepatocytes (85). UA exacerbates lipid metabolism disorders in NAFLD by oxidatively modifying low-density lipoprotein cholesterol and TG synthesis (86). Furthermore, UA facilitates the conversion of fructose to fructose 1-phosphate by activating fructokinase in hepatocytes, leading to fat accumulation through gluconeogenesis (87). Mitochondrial OS induced by UA inhibits aconitase in the Krebs cycle, causing citric acid buildup and stimulating enzymes involved in fatty acid synthesis, ultimately promoting *de novo* lipogenesis (72). UA significantly up-regulates the expression of miR-149-5p in hepatocytes, and FGF21, a downstream target of miR-149-5p and closely related to lipid metabolism, whose deficiency can lead to hepatic steatosis, so that uric acid aggravated hepatic fat accumulation through the miR-149-5p/FGF21 axis (67). UA upregulates the expression of lipogenic genes in hepatocytes via the ROS/JNK//AP-1 pathway, increasing triglyceride levels in HepG2 cells and leading to the accumulation of intracellular fat in hepatocytes (88).

### 3.4 Insulin resistance

NAFLD is closely linked to IR, with some considering NAFLD as a hepatic manifestation of IR (89, 90). Obesity is associated with the development of chronic low-grade inflammation, a condition exacerbated by the expansion of adipose tissue (91, 92). The inflammatory characteristics of adipose tissue enhance cytokine production, which in turn contributes to the development of IR (93, 94). In the state of IR, there is an upregulation of lipolysis in adipose tissue, resulting in an increased influx of free fatty acids to the liver (95). Concurrently, hyperinsulinemia stimulates lipogenesis in hepatocytes (96). These metabolic alterations culminate in lipid accumulation within the liver, leading to an increase in intracellular lipid peroxidation products and cytotoxic agents (97). Ultimately, these processes contribute to the onset and progression of NAFLD.The concentration of SUA was found to be independently related with IR (98). UA can inhibit insulin signaling pathways and induce IR by various mechanisms, including activation of the NLRP3 inflammasome and inhibition of IRS1/Akt pathway (72, 99). Elevated SUA levels cause the deposition of urate crystals in pancreatic islets, impairing pancreatic β-cell function and worsening IR (100). Reduced activity of endothelial nitric oxide synthase is also implicated in increased IR in individuals with hyperuricemia (17). Through the aforementioned mechanisms, UA intensifies the level of IR within the body and facilitates the advancement of NAFLD.

## 4 Potential benefits of SUA-lowering therapy in treating NAFLD

Despite the increasing global prevalence of NAFLD, there remain no FDA-approved pharmacological treatments specifically designed to address this condition, largely due to its intricate pathogenesis and multifactorial nature (101). Currently, lifestyle modifications, including substantial weight loss achieved through a low-calorie diet and increased physical activity, are regarded as the primary interventions for both the prevention and management of NAFLD, as weight reduction is correlated with a decrease in liver fat and may facilitate the reversal of disease progression (102). Bariatric surgery has the potential to decrease hepatic steatosis in obese patients with NAFLD (103). Nevertheless, it is important to note that NAFLD is not considered a valid indication for bariatric surgery (104). In terms of pharmacotherapy, the European and American Association for the Study of the Liver recommends the administration of vitamin E and pioglitazone exclusively for select patients diagnosed with NAFLD (105). More current research hotspots regarding therapeutic agents for NAFLD are mainly focused on drug selection against different metabolic targets (106). Given the significant relationship between SUA levels and NAFLD development, researchers have now recognized that SUA-lowering therapy may have a valuable contribution for improving NAFLD outcomes. XO inhibitors like febuxostat and allopurinol are commonly used to lower SUA levels and have shown promise in improving NAFLD in various studies.

Allopurinol, when administered at a daily dose of 100 mg to patients with hyperuricemia, has been found to lead to a significant reduction in the hepatic controlled attenuation parameter score after a three-month period (107). This medication has demonstrated the ability to decrease TG content in HepG2 cells and in the livers of NAFLD mice by inhibiting XO activity (108, 109). Moreover, allopurinol has shown efficacy in reducing histopathological scores and levels of interleukin-1 (IL-1) and IL-2 immunoexpression in the livers of NAFLD rats (110). By inhibiting NRLP3 inflammasome activation, allopurinol has been observed to mitigate hepatic steatosis and IR by lowering SUA levels (78). In diabetic rats, allopurinol has been found to improve hepatic OS and liver injury through the activation of the Nrf2/p62 pathway (111), as well as to reduce hepatic inflammation and lipid accumulation by decreasing hepatic thioredoxin levels (112). Additionally, allopurinol has been demonstrated to enhance fatty acid beta-oxidation in mouse livers and alleviate high fructose diet-induced hepatic steatosis in diabetic rats by modulating inflammation, lipid metabolism, and endoplasmic reticulum stress pathways (113, 114).

On the other hand, febuxostat has demonstrated a more favorable hepatic safety profile in gout patients with NAFLD (115). Treatment with febuxostat for 24 weeks has resulted in reduced SUA levels, as well as decreased levels of aspartate aminotransferase and alanine aminotransferase in NAFLD patients with hyperuricemia (116). In a nonalcoholic steatohepatitis (NASH) mouse model, febuxostat significantly lowered hepatic XO activity and UA levels, leading to improvements in IR, lipid peroxidation, and the accumulation of classically activated M1-like macrophages in the liver (116). Furthermore, febuxostat has been shown to reduce fat accumulation and ROS in HepG2 cells and NASH mice by downregulating the expression of NLRP3/caspase-1/IL-18/IL-1β and improving IR (117). Administration of febuxostat has also been found to normalize fatty acid oxidation-related genes, collagen deposition, fibrotic changes, lipid peroxidation, and inflammatory cytokine expressions in NASH mice (118).

While both XO inhibitors demonstrated comparable effects in lowering UA levels in the bloodstream, febuxostat exhibited a significant reduction in hepatic UA levels and XO activity in a NASH model in mice, a response not observed with allopurinol (116). This decrease in hepatic UA levels and XO activity was associated with a more pronounced prevention of specific NASH characteristics, including IR, lipid peroxidation, the aggregation of classically activated M1-like macrophages, and hepatic inflammation (116). These findings suggest that febuxostat may possess greater potential for ameliorating NAFLD in patients suffering from hyperuricemia.

## 5 Limitations

Currently, although advancements have been made in understanding the relationship between SUA levels and NAFLD and proposed a new direction and goal for solving the multifactorial problem of fatty liver, this area of research still faces several limitations. There is a pressing need for more fundamental experimental studies to elucidate the mechanisms through which SUA influences the development of NAFLD, particularly those mechanisms that are directly implicated in the pathogenesis of NAFLD, and the strengths and potential limitations of SUA and its direct association with OS. Observational studies investigating the effects of SUA-lowering therapies on NAFLD have primarily been conducted in preclinical settings or among specific populations with hyperuricemia. This is largely attributable to the insufficient recognition and valuation of SUA-lowering agents in clinical practice for the management of NAFLD, and SUA-lowering therapies on NAFLD is necessitated further investigation into their efficacy and safety, while also focusing on its effects on body weight, glucose and lipid metabolism, and liver tissue pathology. Furthermore, there is a notable absence of research conclusions regarding the use of SUA-lowering therapies for the prevention of NAFLD, as well as the impact of such treatments on NAFLD population without hyperuricemia. To address these gaps, more extensive, long-term, multi-center clinical studies are required to assess the potential benefits of these interventions.

## 6 Conclusion

In summary, there is a correlation between SUA and NAFLD, with SUA potentially exacerbating NAFLD through various pathways such as OS, inflammatory responses, lipid metabolism disturbances, and IR. Research has explored the potential benefits of SUA-lowering interventions in improving NAFLD. Given the global prevalence of NAFLD and the current limitations in treatment options, identifying novel therapeutic targets for NAFLD is imperative. Targeting XO inhibition as a SUA-lowering therapy may represent a promising avenue for future NAFLD management.
